# Supplementary material for: Risk and clinical predictors of osteoporotic fracture in East Asian patients with chronic obstructive pulmonary disease: a population-based cohort study
Source: PeerJ. 2016 Oct 27;4:e2634. doi: 10.7717/peerj.2634 (PMC5088616; doi:10.7717/peerj.2634)
Supplement: Table S1 [file peerj-04-2634-s001.docx]

Supplement Table 1. ICD-9-CM codes used in this study

| Disease | ICD-9 Code |
| --- | --- |
| **Osteoporotic fracture [= (733.0x + 733.1x) after excluding ((733.1 + (198.5, 588.0, or 588.1))]** | |
| Osteoporosis | 733.0 |
| Osteoporosis, unspecified | 733.00 |
| Senile osteoporosis | 733.01 |
| Idiopathic osteoporosis | 733.02 |
| Disuse osteoporosis | 733.03 |
| Other osteoporosis | 733.09 |
| Pathologic fracture | 733.1 |
| Pathologic fracture, unspecified site | 733.10 |
| Pathologic fracture of humerus | 733.11 |
| Pathologic fracture of distal radius and ulna | 733.12 |
| Pathologic fracture of vertebrae | 733.13 |
| Pathologic fracture of neck of femur | 733.14 |
| Pathologic fracture of other specified part of femur | 733.15 |
| Pathologic fracture of tibia or fibula | 733.16 |
| Pathologic fracture of other specified site | 733.19 |
| **Pathologic fractures due to cancer metastases, renal osteodystrophy or secondary hyperparathyroidism were excluded** | |
| Secondary malignant neoplasm of bone and bone marrow | 198.5 |
| Renal osteodystrophy | 588.0 |
| Secondary hyperparathyroidism | 588.81 |
| **Comorbidities** | |
| Rheumatoid arthritis | 720.0 714.0 714.1 714.2 714.3x 714.4 714.81 |
| Diabetes mellitus | 250.x |
| Hypertension | 401.x~405.x |
| Osteoporosis | 733.0 |
| Dyslipidemia | 272.x |
| Coronary heart disease | 410.x~414.x |
| Renal failure | 403.x, 404.x, 582.x, 583.x, 585.x, 586.x, 588.x |
| Chronic kidney disease | 250.4, 274.1, 403.01, 403.11, 403.91, 404.02, 404.12, 404.92, 404.03, 404.13, 404.93, 440.1, 585.x, 586.x, 588.8, 588.9 |
| Liver disease | 456.x, 571.x, 572.x |
| Stroke | 430.x~438.x |
| Dementia | 290.x, 797.x |
