# Supplementary figures and images for: Risk and clinical predictors of osteoporotic fracture in East Asian patients with chronic obstructive pulmonary disease: a population-based cohort study
Source: PeerJ. 2016 Oct 27;4:e2634. doi: 10.7717/peerj.2634 (PMC5088616; doi:10.7717/peerj.2634)

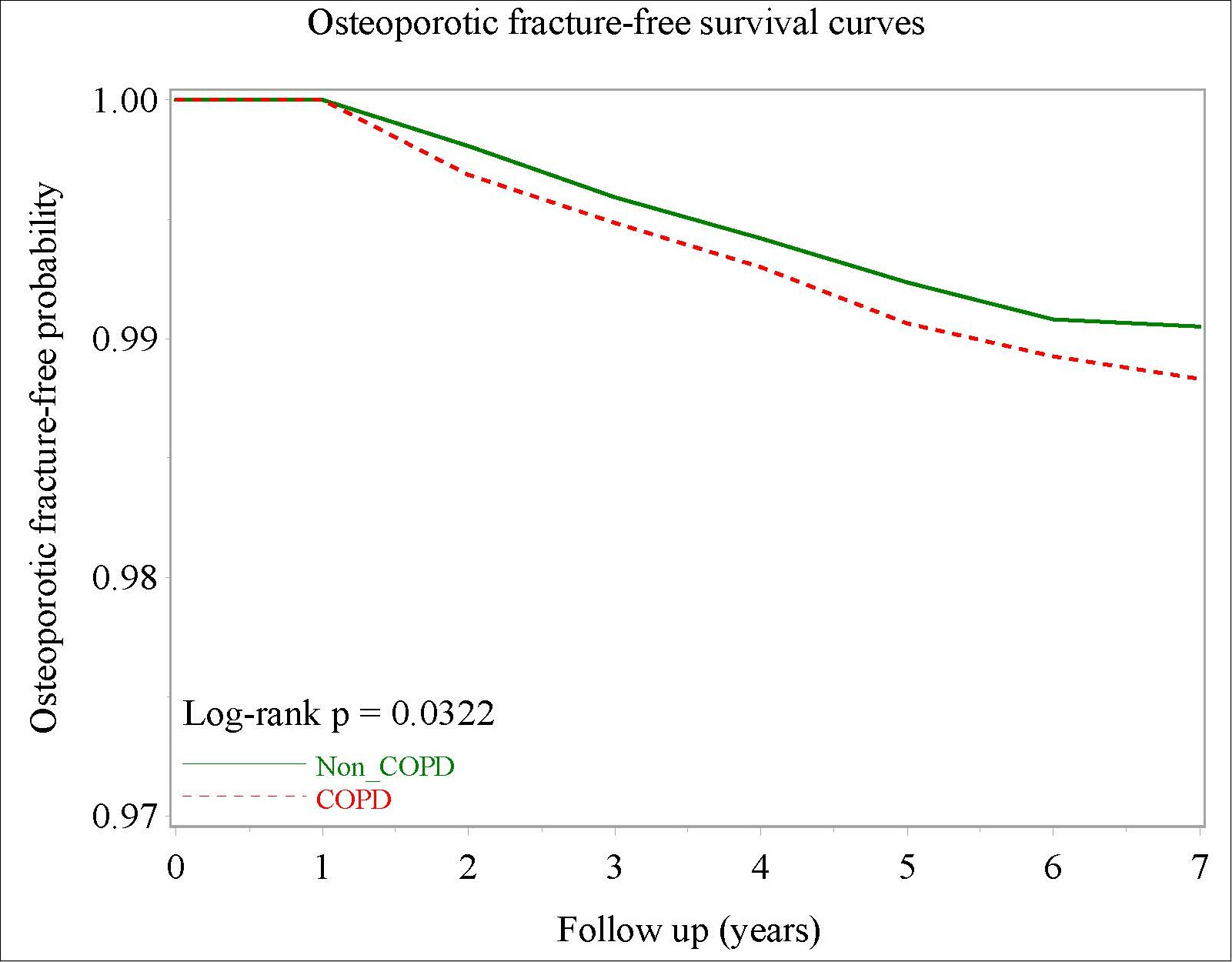

Supplement: Figure S1 [file peerj-04-2634-s002.png]
